# Delayed traumatic aortic valve perforation after blunt chest trauma

**DOI:** 10.1186/s40792-024-01837-6

**Published:** 2024-02-14

**Authors:** Kazuki Noda, Yosuke Takahashi, Akimasa Morisaki, Yoshito Sakon, Kenta Nishiya, Goki Inno, Yukihiro Nishimoto, Yosuke Sumii, Munehide Nagao, Toshihiko Shibata

**Affiliations:** Department of Cardiovascular Surgery, Osaka Metropolitan University Graduate School of Medicine, 1-4-3, Asahichou, Abenoku, Osaka 545-8585 Japan

**Keywords:** Blunt chest trauma, Traumatic aortic valve injury, Delayed aortic valve regurgitation

## Abstract

**Background:**

Aortic valve perforation is a rare complication of blunt chest trauma. We report a case of delayed aortic insufficiency presenting several months after trauma.

**Case presentation:**

A 17-year-old male presented to the emergency department with traumatic brain injuries and blunt chest trauma, but no evidence of cardiac injuries. Three months later, he developed acute heart failure due to severe aortic valve regurgitation with left ventricular dysfunction. A sizable tear in the right coronary cusp caused aortic insufficiency. He was treated successfully by surgical replacement with an aortic bioprosthesis.

**Conclusion:**

We reported a successful surgical case of valve replacement for delayed aortic valve perforation. Delayed valve perforation should be kept in mind after blunt chest trauma.

**Supplementary Information:**

The online version contains supplementary material available at 10.1186/s40792-024-01837-6.

## Background

Aortic valve perforation is a rare complication of blunt chest trauma which can lead to acute aortic regurgitation (AR). Although the clinical course of traumatic AR generally follows a rapid progression, previous reports have described a delayed onset of AR [1, 2]. This report discusses a surgical case of severe AR due to delayed aortic valve perforation presenting three months after blunt chest trauma.

## Case presentation

An otherwise healthy 17-year-old male, with a height of 178 cm, a weight of 60 kg and a body surface area (BSA) of 1.75 m^2^, presented to the emergency department with a traumatic brain contusion, subarachnoid hemorrhage, multiple bone fractures, and blunt chest trauma following a motorcycle collision. Computed tomography and transthoracic echocardiography (TTE) revealed no finding of aortic dissection and aortic valve regurgitation. The patient was treated at the department of neurosurgery and successfully recovered from his brain injuries. The patient was transferred to a rehabilitation hospital 44 days later, with no murmur detected at that time.

**Fig. 1:**
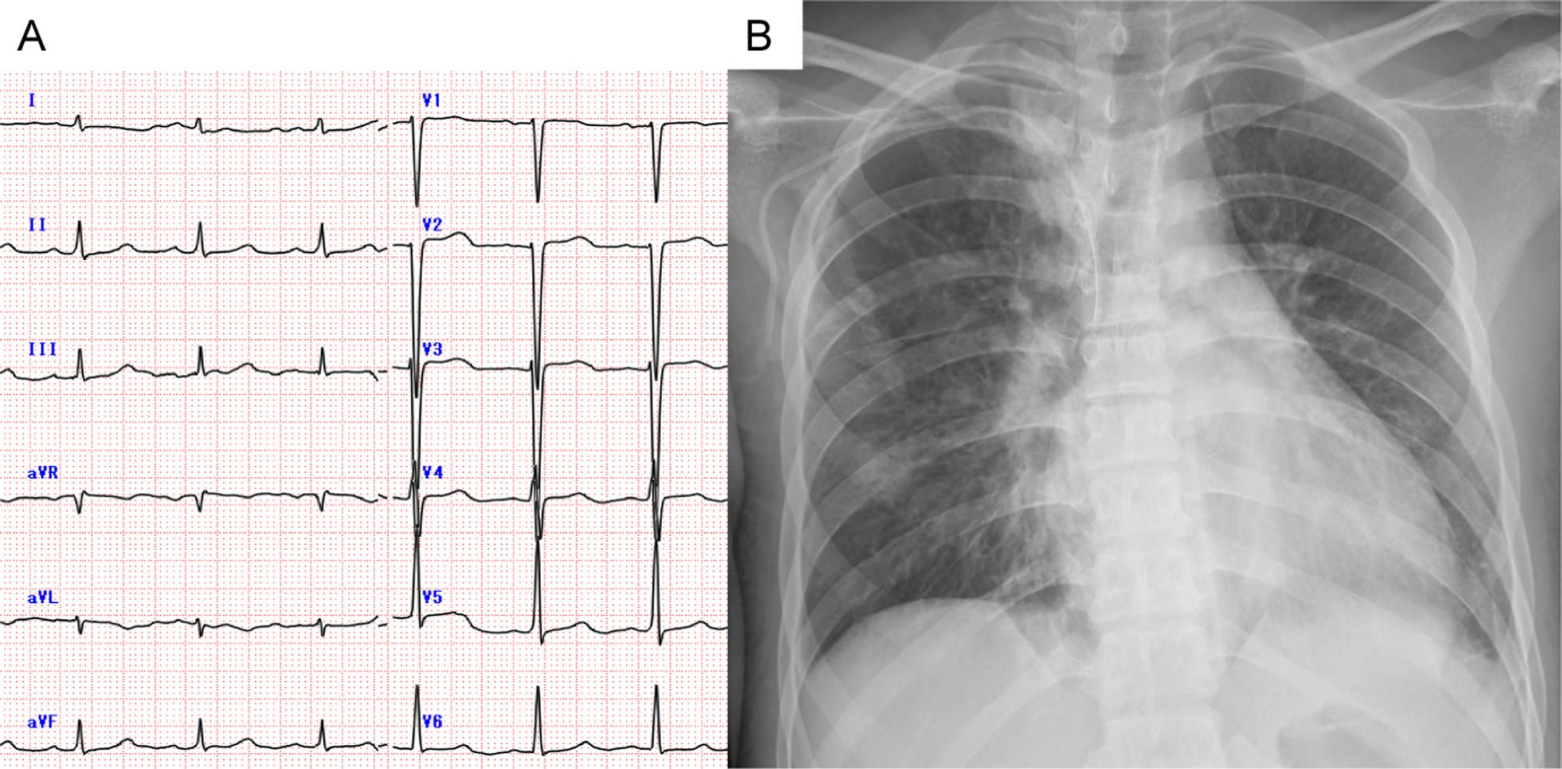
**A** Electrocardiogram showed normal sinus rhythm without ST changes. **B** Chest X-ray with a cardiothoracic ratio of 57%, suggestive of mild cardiomegaly

**Fig. 2:**
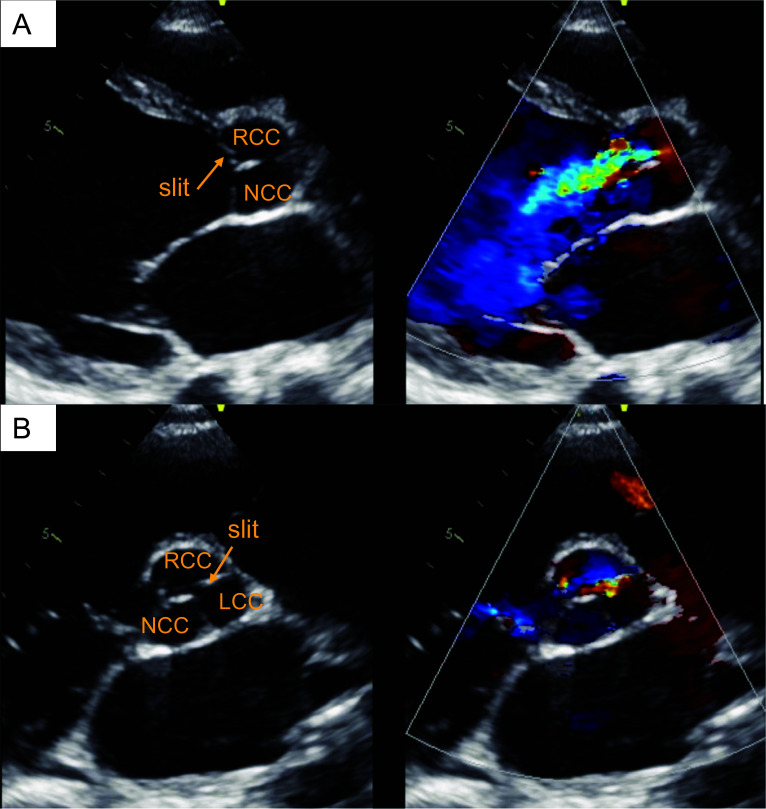
Transthoracic echocardiography in parasternal long-axis view (**A**) and short-axis view (**B**) showed severe aortic valve regurgitation via a slit in the right coronary cusp. *RCC* right coronary cusp, *NCC* noncoronary cusp, *LCC* left coronary cusp

Three months later, the patient had acute heart failure caused by AR after ventriculo-peritoneal shunt operation for hydrocephalus. One month after, he was transferred to our department due to a surgical indication for AR. Upon physical examination, he was bedridden with a grade 4/6 diastolic murmur at the left lower sternal border. His electrocardiogram was essentially normal (Fig. 1A). Chest X-ray revealed an increased cardiothoracic index and pulmonary congestion with a cardiothoracic ratio of 57% (Fig. 1B). TTE revealed left ventricular (LV) dilatation (LV end-diastolic diameter of 64 mm), diffuse LV hypokinesis (LV ejection fraction of 27%), and severe AR through a slit in the right coronary cusp (RCC) into the anterior leaflet of mitral valve (Fig. 2, Additional file 1: Video S1). No vegetation was seen. Laboratory data revealed evidence of heart failure with increased N-terminal pro-brain natriuretic peptide (NT-proBNP) (7884 pg/ml), and hypothyroidism due to hypopituitarism with low free thyroxine (FT4) (0.09 ng/dl) and low thyroid stimulating hormone (TSH) (2.61 μIU/ml).

**Fig. 3:**
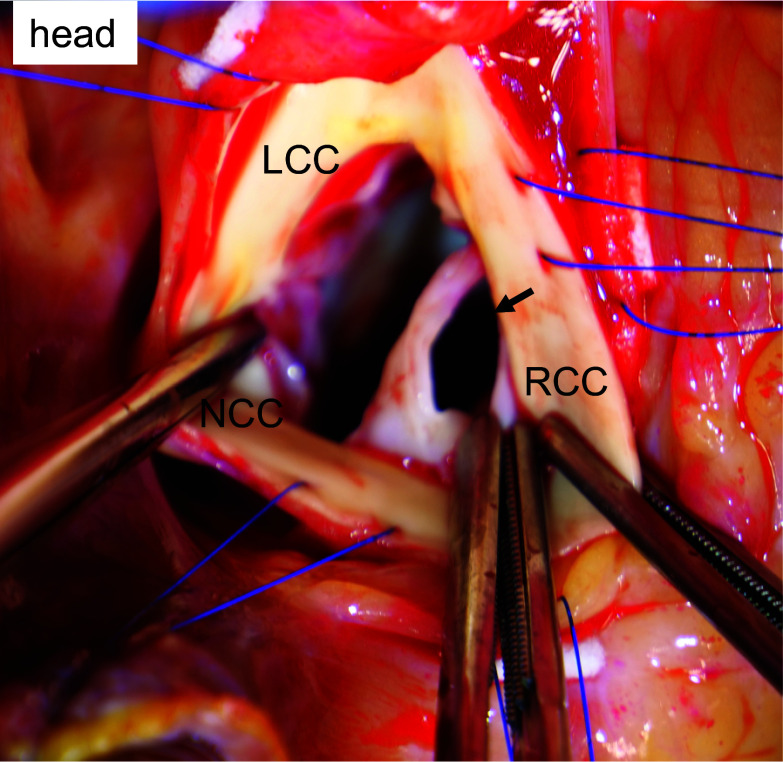
Upon intraoperative inspection of the aortic valve, there was a large perforation (black arrow) of the right coronary cusp. *RCC* right coronary cusp, *NCC* noncoronary cusp, *LCC* left coronary cusp

Despite the uncorrected hypothyroidism, urgent aortic valve replacement was indicated because of the severity of his symptoms. After median sternotomy, aortic cross-clamping, and cardioplegic arrest, the ascending aorta was opened on total cardiopulmonary bypass. Intraoperative inspection of the aortic valve revealed a traumatic tear in the RCC (Fig. 3; Additional file 2: Video S2), with no evidence of endocarditis or calcification. The valve was replaced with a 21-mm INSPIRIS RESILIA (Edwards Lifesciences LLC, Irvine, CA, USA) bioprosthesis with aortic root patch enlargement for the small sinotubular junction. Pathologic analysis revealed myxomatous degeneration of the leaflets without findings suggestive of inflammation or malignancy. The postoperative course was uneventful, and the patient was referred to the rehabilitation hospital 26 days after surgery. Six months later, the patient was ambulatory, and TTE showed a normally functioning aortic valve prosthesis and preserved LV function (LV ejection fraction of 58%).

## Discussion

Blunt chest trauma is common after motorcycle accidents and is the second leading cause of death after brain injury [3]. Isolated valvular injury is rare [4], with a reported incidence of 5% after blunt cardiac injury [5]. Aortic valve perforation is the most frequently reported valvular injury, characterized by subsequent aortic insufficiency of sudden onset, excluding other causes of aortic incompetence [6].

The available information regarding traumatic aortic valvular injury is scarce and mainly derived from case reports. Although the symptoms of aortic valvular injury are often acute [7], these can also present late as in our case. Aortic valvular injury is caused by a sudden increase in intra-aortic pressure, especially during early diastole, when the transaortic gradient is maximal. The initial small tear or rupture by this high pressure transmitted toward the closed aortic valve might have progressively extended due to hemodynamic stress. As the valve cusp separated, AR progressed with a compensatory increase in the force of ejection and consequently increased hemodynamic stress to the valve cusp [8]. Furthermore, the RCC is not the most commonly damaged location. This is because diastolic hemodynamic stress occurs over the right and left coronary cusps during blood flow to the coronary arteries, but not over the noncoronary cusp, which is the most commonly involved [1]. Therefore, the delayed aortic valve perforation in the RCC in our case was very unusual.

This patient had rapidly progressive LV dysfunction with reduced ejection fraction after developing aortic insufficiency. Although LV dysfunction was primarily attributed to aortic insufficiency, overt hypothyroidism has been also associated with decreased cardiac contractility and ejection fraction [9], and uncorrected hypothyroidism can also cause LV dysfunction. However, in a previous review, there was no significant difference in the rate of major adverse postoperative cardiac events, including mortality, based on thyroid status [10]. In this case, because it would take a long time to achieve a euthyroid status, the aortic valve operation was prioritized.

The indication for valve replacement or repair depends on the extent of cusp injury. Some patients have reported good results after valvuloplasty [11], but its long-term outcomes are unknown. The aortic leaflets can appear undamaged regardless of pathologically abnormality, and this can cause valvular insufficiency after valvuloplasty [8]. Also, due to inadequate control of hydrocephalus, the patient’s preoperative level of consciousness was impaired, raising the possibility of the need for additional neurosurgical procedures after valve surgery. Thus, in this case of deteriorated LV function, after considering the risk of cerebral hemorrhage, valve replacement was performed using a prosthetic tissue valve.

## Conclusions

We reported a successful surgical case of valve replacement for delayed aortic valve perforation which developed three months after blunt chest. In cases of high-energy blunt chest trauma, delayed valve insufficiency should be monitored. Should valvular dysfunction occur, the timing of the surgical intervention is crucial.

### Supplementary Information

**Additional file 1: Video S1.** Transthoracic echocardiography in parasternal long-axis view (A) and short-axis view (B) showed severe aortic valve regurgitation with left ventricular dysfunction.

**Additional file 2: Video S2.** Intraoperative video of the large perforation of the right coronary cusp.

## Data Availability

The datasets used and/or analyzed during the current study are available from the corresponding author on reasonable request.

## Abbreviations

AR: Aortic regurgitation
BSA: Body surface area
TTE: Transthoracic echocardiography
LV: Left ventricular
RCC: Right coronary cusp
NT-proBNP: N-terminal pro-brain natriuretic peptide
FT4: Free thyroxine
TSH: Thyroid stimulating hormone
